# Migraine Characteristics Among Smokers and Non-Smokers: A Cross-Sectional Survey in Saudi Arabia

**DOI:** 10.3390/healthcare14020207

**Published:** 2026-01-14

**Authors:** Abdullah Alsabaani, Mona Hussain Aldukain, Ali Hussain Aldukain, Roaa Al Murayyi, Shahad Ali Alshehri, Shuruq Abdullah M. Alqahtani, Omair Mohammed O. Alshahrani, Abdulmohsin Mohammed S. Alzuhairi, Syed Esam Mahmood

**Affiliations:** 1 Department of Family and Community Medicine, College of Medicine, King Khalid University, Abha 61421, Saudi Arabia; aalsabaani@kku.edu.sa; 2 College of Medicine, King Khalid University, Abha 61421, Saudi Arabia

**Keywords:** migraine, tobacco, smoking behaviour, Saudi Arabia

## Abstract

**Background:** Migraine is a prevalent neurological disorder associated with significant morbidity and social burden. Although various triggers for migraine have been identified, the relationship between smoking and migraine remains unclear. This study aimed to compare migraine characteristics between people with and without smoking in Saudi Arabia. **Methods:** A cross-sectional study using an online survey tool had been conducted in Saudi Arabia. The survey assessed migraine characteristics, smoking behaviour, demographics, and comorbidities. Statistical analyzes were performed to investigate the occurrence of migraine, smoking behaviour, and demographic factors. Descriptive statistics summarized the data, with various statistical tests employed to compare variables between groups. **Results:** A total of 229 participants were included in the study, with a majority being young adults (48.47%), predominantly females (66.81%), and holding a bachelor’s degree (63.32%). The study found that 19.2% of individuals with migraine were current smokers, with an average smoking duration of 9.7 years. While some reported relief from migraine pain, others experienced increased pain intensity or frequency. No significant differences were found in migraine characteristics between smokers and non-smokers, but younger individuals and males with migraine were more likely to smoke. The study highlights the complex relationship between smoking and migraine, with varying effects on individuals. **Conclusions:** The study underscores the lack of significant differences in migraine characteristics between smokers and non-smokers, suggesting that smoking does not play a pivotal role in the clinical presentation of migraines. This insight prompts a shift in research focus towards other potential contributors to migraines, such as genetic predispositions, environmental factors, and comorbidities. Understanding these associations can inform public health strategies aimed at alleviating migraine-related burdens.

## 1. Introduction

More than a billion people worldwide suffer from migraine, a recurrent neurological disorder marked by excruciating headaches that are frequently accompanied by other incapacitating symptoms [1]. As such, it is one of the most common and burdensome medical conditions. Various factors have been implicated as triggers for migraine episodes, including stress, hormonal fluctuations, specific foods, and environmental stimuli [2]. Among these triggers, tobacco smoking has garnered considerable attention as a potential precipitating factor for acute migraine attacks. Although smoking is linked to many health risks like heart disease, cancer, and breathing problems, the connection between smoking and migraines is still not fully understood [3,4,5].

In 2023, a study by Kobus et al. found a significant link between maternal tobacco smoking during pregnancy and increased migraine risk in adults aged 18–65 [6]. Previous research, including a 2009 study by López-Mesonero et al., indicated that smoking may trigger migraine attacks, with higher smoking rates among migraine sufferers [7]. Rozen’s study also noted a connection between cigarette smoking and cranial autonomic symptoms in migraine patients [8]. Additionally, Weinberger et al. (2023) reported a higher prevalence of smoking among migraine patients, suggesting it may worsen migraine-related complications like stroke [9]. Sarker et al. further confirmed that migraine patients had higher exposure to both smoked and smokeless tobacco compared to controls, even after accounting for other risk factors [10].

Research indicates that the prevalence of migraines varies by location. The prevalence in Khobar City’s Thuqbah district has been estimated to be 5% [11]. The incidence increased to 12.3% after moving to Aseer in the southern portion of the nation [12]. In Taif, located in western Saudi Arabia, the prevalence was very high, with 78.5% of the sample reporting migraine headaches. This was a cross-sectional study carried out on 354 individuals using the International Headache Society (IHS) criteria [13]. In the eastern province, about 40% of survey participants reported having migraine headaches [14]. A recent comprehensive study conducted throughout several parts of the Saudi Kingdom revealed the prevalence of migraine to be 27.4% [15].

However, limited investigation has been conducted in Saudi Arabia to explore the clinical characteristics of migraine among smokers and non-smokers. With the prevalence of smoking remaining a significant public health concern in Saudi Arabia [16], understanding its potential impact on migraine not only has implications for individual patient care but also for broader public health initiatives [17] aimed at reducing the burden of migraine and related comorbidities. Given the unique cultural, environmental, and genetic factors at play in Saudi Arabia, investigating the relationship between smoking and migraine within this context has the potential to yield insights that may not be fully captured in studies conducted in other regions. By examining the characteristics of migraine in both smokers and non-smokers within the Saudi Arabian population, our study aimed to shed light on the impact of smoking on migraine and elucidate its role in precipitating or exacerbating migraine attacks. This endeavor held potential significance for healthcare professionals in developing tailored management strategies for patients presenting with migraines and smoking habits. Furthermore, the potential to inform evidence-based interventions and policies aimed at reducing smoking prevalence and improving migraine management outcomes in the region.

## 2. Materials and Methods

### 2.1. Study Design

This study employed a cross-sectional survey design in Saudi Arabia from December 2023 to March 2024. The Research Question was: What are the differences in the characteristics of migraine (including duration, frequency, and type of pain) between smokers and non-smokers in Saudi Arabia?

### 2.2. Study Participants

Participants complaining of migraine were recruited from various regions of Saudi Arabia using convenience sampling methods. Individuals aged 18 years and above were eligible to participate. Patients who were unable to complete the surveys due to cognitive impairment or language barriers or with a history of chronic illnesses that might significantly impact migraine characteristics, such as neurological disorders or psychiatric conditions were excluded from our study. Participants suffering from migraine were divided into two groups based on their smoking status: smokers (current smokers) and non-smokers (those who had never smoked or were former smokers). Definitions used were: Current smoker: daily or occasional use at the time of survey. Former smoker: previously smoked but not currently. Never smoker: never used any tobacco product.

### 2.3. Sample Size

To determine the appropriate sample size, we conducted a pilot study that included 30 migraine patients, of whom 8 were smokers. Based on this pilot, we found that the proportion of participants reporting that migraine attacks impacted their daily activities was 20% among non-smokers and 45% among smokers. These assumptions informed the sample size calculation using G*Power software (www.psychologie.hhu.de, accessed on 14 September 2025), targeting a power of 95% and an alpha error of 0.05, with a 1:4 ratio of smokers to non-smokers. The minimum required sample size based on these parameters was 195 participants.

### 2.4. Data Collection Tool

A structured survey was developed for data collection, based on a review of existing literature and validated assessment tools where applicable [7,10,11,12,13,14]. The questionnaire assessed current smoking status and active tobacco use only. Exposure to passive (second-hand) smoking, including household or childhood exposure, was not evaluated.

The survey consisted of several sections:

1. Demographic information: Age, gender, education level, occupation, and residential region.
2. Migraine characteristics: Participants were queried about their migraine history, including frequency, duration, intensity of migraine attacks, type of pain, associated symptoms, and types of auras.
3. Co-morbidities: Information regarding any co-existing medical conditions, such as hypertension, diabetes, cardiac or other chronic diseases, was also collected.
4. Smoking behaviour: Participants were asked about their smoking status (current smoker, former smoker and non-smoker), frequency of smoking, duration of smoking (in years), and average number of cigarettes smoked per day.

### 2.5. Data Collection Method

The survey was administered online. Participants were recruited through various channels of different social media platforms. To ensure confidentiality and data security, participants were provided with the survey links and were assured that their responses would remain anonymous and confidential. The survey was accessible via desktop computers, laptops, tablets, and smartphones, allowing participants to complete it at their convenience. Participants were informed about the purpose of the study and provided with informed consent prior to participation. Confidentiality of responses was ensured, and participants were assured that their data would be used for research purposes only.

### 2.6. Ethical Considerations

This study was approved by the Institutional Review Board (IRB) of [ECM#2023-3313] from King Khalid University (HAPO-06-B-001). Informed consent was obtained from all participants prior to data collection.

### 2.7. Data Quality Assurance

To maintain data quality and integrity, several measures were implemented throughout the data collection process. These included validation checks within the online survey platform to minimize data entry errors, monitoring of response completeness, and periodic review of response patterns.

### 2.8. Statistical Analysis

Descriptive statistics were used to summarize demographic characteristics, migraine characteristics, co-morbidities, and smoking behaviour of the participants. Demographic variables and comorbidities were compared between participants suffering from migraine and participants without migraine. Chi-square tests were employed to compare categorical variables between smokers and non-smokers, while *t*-tests or Mann–Whitney U tests were used for continuous variables, depending on the distribution of the data. Statistical analyses were performed using Stata, www.stata.com, accessed on 7 November 2024 (StataCorp LLC, College Station, TX, USA). A *p*-value less than 0.05 was considered statistically significant.

## 3. Results

A total of 229 participants were included. Most participants (48.47%) fall within the 20–29 age group, while smaller percentages are distributed across other age groups, with only 0.44% aged 60–69 years. Most of the sample is female (66.81%), with males making up 33.19%. Almost, one-half of the participants are single (50.66%) or married (45.41%), or divorced (3.93%). A sizable portion of the sample consists of employees (37.12%) and students (33.62%). Over half of the participants (58.95%) earn less than 5000 SAR per month, with fewer earning between 5000 and 15,000 SAR, and only 10.04% earning over 15,000 SAR. Most participants hold a bachelor’s degree (63.32%), followed by those with secondary education (28.82%). A small portion has post-graduate education (5.68%), while very few are having primary education (2.18%, respectively), as shown in Table 1.

**Figure 1:**
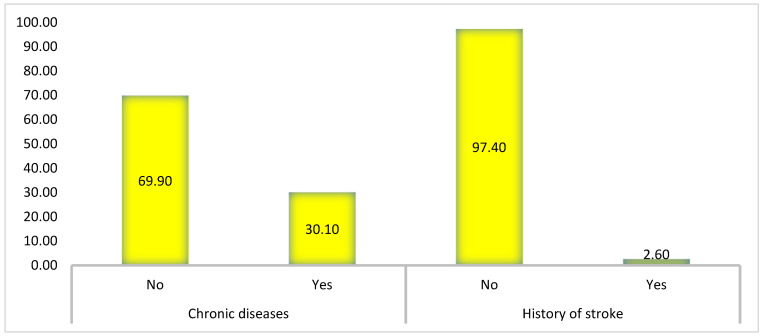
Percentage of chronic diseases and history of stroke in the study population.

Approximately 30.10% of the participants reported having chronic diseases, while the majority (69.90%) indicated that they did not have any chronic conditions. A small fraction of the sample (2.60%) has experienced a stroke, with the vast majority (97.40%) reporting no history of stroke, as shown in Figure 1.

Among participants with migraine who reported smoking, the mean smoking duration was approximately 9.7 years, with a wide range of daily cigarette consumption. Most smokers (48%) reported consuming 1–10 cigarettes per day. Interestingly, a substantial proportion of smokers reported various effects of smoking on migraine as relief of pain (27%), while 23% reported increase intensity of pain and 7% reported increase frequency of attack. This suggests considerable variability in the relationship between smoking behaviour and migraine outcomes among individuals with migraine in this population, as shown as in Table 2.

Table 3 compares migraine characteristics between non-/ex-smokers and current smokers. The mean duration of migraine history, frequency of weekly attacks, and average duration of migraine episodes were comparable between the two groups, with no statistically significant differences observed (*p* > 0.05 for all). More than half of participants in both groups reported that migraine attacks interfered with daily activities, affecting 50.8% of non-/ex-smokers and 52.3% of smokers, with no significant difference between groups (*p* = 0.77).

Regarding pain characteristics, pulsatile pain was the most commonly reported type in both non-/ex-smokers (51.4%) and smokers (56.8%), followed by pressure-type pain, while stabbing pain was less frequent in both groups. No statistically significant association was observed between smoking status and type of migraine pain (*p* = 0.47).

Analysis of associated symptoms showed similar distributions between groups. Increased sensitivity to light and noise was reported by 42.2% of non-/ex-smokers and 52.3% of smokers, while visual disturbances were reported by 26.5% and 27.3%, respectively. Although visual disturbances were slightly more frequent among smokers, the overall pattern of associated symptoms did not demonstrate clinically meaningful differences between smoking groups.

With respect to migraine aura, approximately half of participants in both groups reported no aura, while visual aura was the most common aura type among those who experienced aura. No significant differences were observed in aura patterns between non-/ex-smokers and smokers (*p* = 0.59).

**Figure 2:**
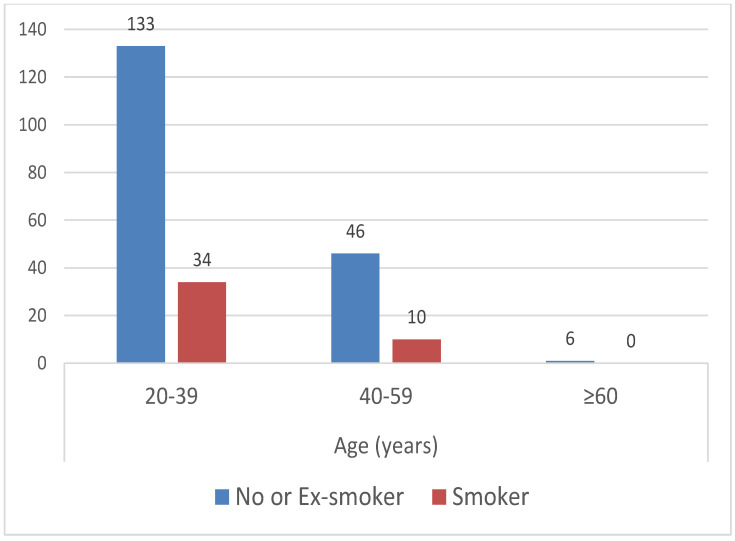
Smoking status by different age groups.

Among participants with migraine, a higher proportion of younger individuals were observed to be smokers compared to non-smokers (Figure 2).

**Figure 3:**
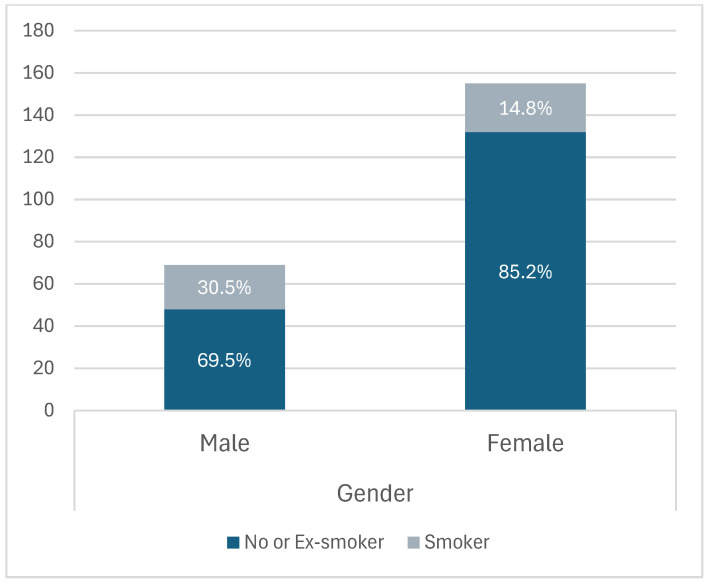
Smoking status by gender.

Furthermore, most of the smokers that suffered from migraine were males (30.5%). (Figure 3).

## 4. Discussion

Our study examined the clinical characteristics of migraine in individuals with and without tobacco smoking habits in Saudi Arabia. The findings provide valuable insights into the epidemiology of migraine and its association with smoking behaviour, enhancing our understanding of potential implications for clinical management and public health interventions.

Our analysis identified significant associations between migraine status and various demographic factors. Notably, a higher prevalence of migraine was found among younger individuals, aligning with established research indicating that migraine onset typically occurs in early adulthood and tends to decrease with age [18]. This age-related trend emphasizes the need to consider developmental and hormonal factors in the pathogenesis and management of migraines. Consistent with previous studies, age emerged as a significant factor influencing migraine prevalence across all age groups [19]. Almost half of the participants are aged 20–29 years, which may limit the generalizability of the findings, as young adults often differ from older individuals in behaviors, health awareness, and physiological responses, and migraine and smoking patterns can vary with age. Additionally, our findings suggest regional disparities in migraine prevalence, with higher rates reported in certain areas of Saudi Arabia. These variations may reflect differences in environmental exposures, socioeconomic conditions, and healthcare access, warranting further investigation [20]. Furthermore, migraine sufferers were more likely to be unemployed and to have moderate income levels compared to non-sufferers. This highlights the potential impact of socioeconomic factors on migraine prevalence and underscores the importance of addressing social determinants of health in migraine management. A related study found that individuals with migraines were more likely to be unemployed than those without migraines [21], likely due to the debilitating nature of migraines, which can lead to missed workdays and reduced productivity.

Migraine Characteristics: Contrary to our expectations, we did not find significant differences in migraine characteristics between smokers and non-smokers. Previous research has suggested that smoking may exacerbate migraine severity and frequency [6,7,8], potentially through nicotine-induced changes in cerebral blood flow, oxidative stress, and inflammation. However, our study may have lacked sufficient power to detect such differences due to the small sample size of smokers with migraines. Future research with larger cohorts is necessary to confirm these findings and explore the underlying mechanisms linking smoking and migraine characteristics.

The majority of participants reported that migraines significantly impacted their daily activities, highlighting the substantial burden of migraine on individuals’ functioning, regardless of smoking behaviour. This concern is supported by various studies indicating that patients with migraines experience a greater burden compared to non-headache patients [22]. A considerable percentage of participants also reported spending time in darkness or isolation due to migraines, further affecting their daily functioning [23]. Additionally, Bigal et al. noted that patients with chronic migraines experience significant impairment in their daily activities, indicating a severe burden from the condition [24].

Our results indicated no significant differences in the type of pain experienced during migraine attacks between non-smokers/ex-smokers and smokers. This suggests that smoking status may not influence the qualitative aspects of migraine pain in this study population. However, existing research indicates a complex relationship between smoking and pain perception. While some studies suggest that smokers may experience more severe pain than non-smokers [25], others report no significant differences in pain intensity [26]. Additionally, research has shown that smokers may have a higher prevalence of chronic pain and more widespread pain compared to non-smokers [27]. It is important to note that the impact of smoking on pain perception can vary depending on the specific condition being studied.

Smoking Characteristics: Among participants with migraines who reported smoking, we observed a range of smoking behaviours and attitudes. The mean duration of smoking and the average number of cigarettes consumed per day were consistent with findings from previous studies [6,7]. A significant proportion of participants also reported exposure to second-hand smoke, which has been linked to increased migraine prevalence and severity. Previous studies have suggested that second-hand smoke, particularly during childhood, may influence migraine development and severity through neurovascular and inflammatory mechanisms. The inability to account for passive smoking may have led to misclassification of true tobacco exposure among non-smokers, potentially attenuating observed differences between groups [6,9,10]. These findings underscore the importance of considering both active and passive smoking exposure when assessing migraine risk and developing targeted smoking cessation interventions.

We also noted a higher proportion of younger individuals and males among smokers compared to non-smokers. These findings may provide insights into the demographic correlates of smoking behaviour among migraine sufferers.

### Limitations and Future Directions

The study presents several limitations that should be acknowledged. Firstly, the cross-sectional methodology employed restricts the ability to establish causality between smoking and migraine, indicating a need for longitudinal studies to better understand the temporal relationship between these variables. Additionally, the reliance on self-reported data introduces the potential for recall bias, particularly regarding participants’ smoking habits and migraine symptoms.

Additionally, the small sample size was a factor affecting the precision of the study results. A larger number of participants would enhance statistical power and potentially broaden the scope of statistical interpretation between smokers and non-smokers.

To enhance the validity of future findings, it is recommended that objective assessments of both smoking status and migraine characteristics be incorporated. Moreover, the use of convenience sampling and the specific demographics of the population studied may limit the generalizability of the results. Finally, exposure to passive (second-hand) smoking was not assessed, including exposure during childhood, which may be relevant to migraine susceptibility. As a result, some non-smokers may have had significant indirect tobacco exposure, potentially diluting the association between smoking status and migraine characteristics. Another limitation of the current study is the lack of differentiation between various types of smoking (e.g., cigarette, cigar, electronic). The original survey did not account for these distinctions, which may influence the association between smoking and migraine. Future research should incorporate stratification by smoking type to provide a more comprehensive understanding of their respective impacts. The potential impact of smoking cessation on migraine has also not been addressed in survey data. Although some data on aura were collected, the study lacked sufficient power to perform subgroup analyses distinguishing between episodic and chronic migraine, as well as migraine with aura and without aura. Future research should aim to explore these distinctions to better understand their potential differential impacts.

The overrepresentation of young adults in the study sample potentially impacts the observed associations between smoking and migraine. Recognizing this limitation is crucial for interpreting the findings and underscores the need for future research with more age-diverse populations to elucidate the complex interplay between age, smoking, and migraine.

Future research should aim to include a more diverse and representative sample to ensure that the findings can be applied to a broader population.

## 5. Conclusions

Our study sheds light on the relationship between migraine characteristics and tobacco smoking habits among individuals in Saudi Arabia. Although we found no significant differences in migraine characteristics between smokers and non-smokers, our results emphasize the significance of considering demographic and smoking-related factors in migraine assessment and management. To improve migraine outcomes, healthcare professionals should prioritize screening and counseling patients for tobacco use and smoking cessation. Public health initiatives focusing on reducing smoking prevalence and exposure to second-hand smoke may also contribute to alleviating the burden of migraine on individuals and society. Future studies with larger sample sizes, longitudinal approaches, and comprehensive assessments of smoking behaviour and migraine characteristics are necessary to confirm our findings and inform effective prevention and management strategies for migraine patients.

## Figures and Tables

**Table 1:** Socio-Demographic Characteristics of the Study Population (*n* = 229).

Variable	Level	Counts	%
Age in years	16–19	5	2.18
	20–29	111	48.47
	30–39	51	22.27
	40–49	37	16.16
	50–59	24	10.48
	60 or above	1	0.44
Gender	Female	153	66.81
	Male	76	33.19
Marital status	Single	116	50.66
	Married	104	45.41
	Divorced	9	3.93
Occupation	Student	77	33.62
	Retired	16	6.99
	Employee	85	37.12
	Unemployed	44	19.21
	Businessman/woman	4	1.75
	Housewife	3	1.31
Salary	<5000 SAR	135	58.95
	5000–10,000 SAR	48	20.96
	10,000–15,000 SAR	23	10.04
	>15,000	23	10.04
Education	Upto Primary education	5	2.18
	Secondary education	66	28.82
	Bachelor	145	63.32
	Post-graduate education	13	5.68

**Table 2:** Smoking characteristics among participants with migraine.

Factor	Value
N	44
Smoking duration (years), mean (SD)	9.7 (9.2)
Number of cigarettes consumed per day	
1–10	21 (48%)
11–20	14 (31.8%)
21–30	7 (15.9%)
31–40	2 (4.5%)
Effects of smoking on Migraine attacks	
No effect	17 (38.6%)
Decrease frequency of attacks	2 (4.5%)
Increase frequency of attacks	3 (6.8%)
Increase the intensity of the pain	10 (22.7%)
Relief from the pain	12 (27.2%)

**Table 3:** Migraine Characteristics according to their smoking status among study participants, *n* = 229.

Factor	Total 229 (100%)	Non- or Ex-Smoker 185 (80.8%)	Smoker 44 (19.2%)	*p*-Value
For how long have you been experiencing migraine? (months), mean (±SD)	42.4 (±65.4)	52.7 (±81.6)	37.9 (±61.0)	0.39
Number of migraine attacks per week, mean (±SD)	3.7 (±1.7)	3.6 (±1.8)	3.4 (±1.7)	0.51
How long on average does each migraine attack take? (Hours), mean (±SD)	6.3 (±9.6)	6.4 (±9.4)	4.9 (±7.8)	0.47
Do your migraine attacks affect your daily activities? (Yes)	117 (51.1%)	94 (50.8%)	23 (52.3%)	0.77
Type of pain				0.47
Stabbing	18 (7.8%)	13 (7.0%)	5 (11.4%)	
Pressure	91 (39.7%)	77 (41.6%)	14 (31.8%)	
Pulsatile	120 (52.4%)	95 (51.4%)	25 (56.8%)	
Associated symptoms (multiple responses)				
Increase sensitivity to light and noise	101 (44.1%)	78 (42.2%)	23 (52.3%)	0.226
Visual disturbances	61 (26.6%)	49 (26.5%)	12 (27.3%)	0.011
Verbal disturbances	62 (27.1%)	53 (28.6%)	9 (20.5%)	0.273
Confusion	59 (25.8%)	47 (25.4%)	12 (27.3%)	0.064
Main Aura with migraine attacks				0.59
Visual aura	67 (29.3%)	55 (29.7%)	12 (27.3%)	
Sensory aura	35 (15.3%)	27 (14.6%)	8 (18.2%)	
Speech or language impairment	13 (5.7%)	9 (4.9%)	4 (9.1%)	
No aura	114 (49.7%)	94 (50.8%)	20 (45.5%)	

## Data Availability

The raw data supporting the conclusions of this article will be made available by the authors on request.

## Abbreviations

IHS	International Headache Society
IRB	Institutional Review Board
